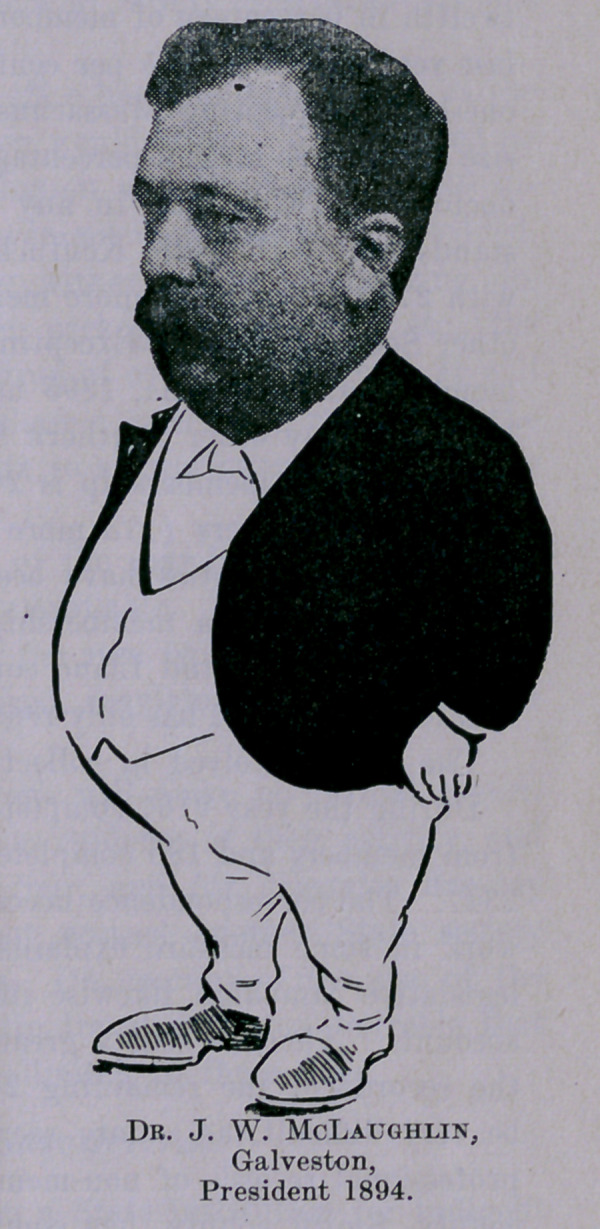# Thirty-seventh Annual Meeting, State Medical Association of Texas, Houston, Texas, April 24, 25, 26, 27, 28, 1905

**Published:** 1905-05

**Authors:** 


### Thirty-seventh Annual Meeting, State Medical Association of Texas, Houston, Texas, April 24, 25, 26, 27, 28, 1905.

[Acknowledgments: To the courtesy of Mr. Hobby, managing editor of the *Houston Post,* I am indebted for the use of most of the pictures in this write-up; and much that follows is clipped from the *Post* and reproduced verbatim. The physicians of Texas deeply appreciate the many courtesies of this progressive newspaper, and the Association unanimously voted a vote of thanks to the *Post* for full and correct reports daily, of this important gathering, the largest assembling of doctors ever held in the South. The meetings were reported by the talented and obliging Colonel Petty, of the *Post.* The other pictures I bought from the proprietor of the *Chronicle.* He could not "loan" them, he said, but would *sell them to me* "below cost." We are indebted to the *Chronicle* for reports.—Dr. Daniel.]

From the *Chronicle:*

"Back home again are the members of the State Medical Association of Texas. From President Daniel and Secretary Chase down to the newest and youngest member of the organization of the physicians of the State there appears to have been an eagerness to get back to Houston for the annual assembling of the doctors, and every member holds a warm spot in his heart for this city, as they feel that the city that cradled the society is entitled to much consideration when the full grown, lusty babe of thirty-seven years ago, in its mature age, seeks to again spend a week within the confines of the Magnolia City. Every seven years since the Association was reorganized in 1869, the doctors have assembled in Houston, but the 1905 session promises to eclipse all of the past meetings in Houston, both in the way of local entertainment and in the amount of interest the members are evidencing.

In addition to the interest pertaining to the session of the House of Delegates and the open session, where all the latest methods in the practice of medicine and surgery are discussed, here is the most lively curiosity to see what the great instrument manufacturing concerns and printing houses are putting out this year.

The main entrance hall and the reading room of Turner Hall have been converted into a miniature exposition. X-ray machines, surgical instruments, all sorts of concoctions and mixtures are on every side. The medical journal men and the book publishers are out in force and in addition to this there has been procured from the University of Texas a most elaborate and interesting exhibit. This includes plaster and wax figures, showing the effects of certain diseases and methods of operation. The physicians are enthusiastic in their praise of this exhibit.

The House of Delegates was assembled for the first session at 8 o'clock last night, but beyond the submission of the message by the president, there was nothing done, and the session of this morning, called to order at 9 o'clock, did not bring about any important results or the appointment of any of the committees. A half dozen district reports have been submitted by the various district representatives. An adjournment was taken shortly after 10 o'clock until 2 o'clock this afternoon, when more district reports were received. These reports show the Association to be making a splendid growth in membership throughout the State.

A few moments after 10 o'clock Dr. S. C. Red, chairman of the local arrangement committee, called the main assembly to order in the large auditorium at Turner Hall, and more than 300 visiting

DR. J. E. GILCREEST, GAINESVILLE, TEXAS,

President-Elect State Medical Association of Texas.

F. E. DANIEL, M. D., AUSTIN, TEXAS,

Retiring President State Medical Association of Texas.

physicians and many Houstonians arose to their feet while Dr. William Hayne Leavell asked divine guidance at the opening of this, the thirty-seventh session of the State Medical Association of Texas.

Mayor Jackson was not present to deliver an address on behalf of the city, as was indicated as the next number on the program, and Hon. C. R. Wharton was introduced by Chairman Red, and delivered a beautiful and eloquent address. Dr. E. N. Gray, president of the Harris County Medical Society, welcomed the Association, as did Dr. S. C. Red, ex-president and chairman of the committee of arrangements, in appropriate and elegant language.

President F. E. Daniel, of Austin, was then introduced by Chairman Red, and as he was announced as the courtly gentleman and splendid orator, he was roundly applauded by the members of the Association. He spoke of the warmth of the welcome extended, and referred to the fact that Houston was the home of the society. He spoke of the city's industrial prominence and said that, while Houston was great, she was destined to be far greater, and he depicted a dazzling future. He said the captains of industry here had the "get there" spirit that wins. He said the doctors would make themselves at home, and he knew they would enjoy every minute spent in this city.

Following the addresses, the meeting was by Dr. Red turned over to President Daniel, and he at once got the doctors down to work.

The President's message and recommendations were then read in the House of Delegates; meanwhile the Section of Surgery was called. There were twenty-two papers in this section, some by noted men. I have not room to report the sections.

THE PRESIDENT'S MESSAGE.

GENTLEMEN: I congratulate you on the continued prosperity of our Association, and the steady interest and zeal in the cause of organized legitimate medicine. I congratulate you upon this great gathering of members, which evidences an unabated interest in it. And I thank you, one and all, for the loyal support and cordial cooperation you have given me during my brief administration as your President.

MEDICAL LEGISLATION.

Our efforts to secure legislation in the interest of science and public health have been earnest and sustained, but unsuccessful. This is not the time nor place to lay before you an account of the

MRS. F. E. DANIEL, "THE MASCOT"

State Medical Association of Texas.

work done by your committee on public policy and legislation to secure the passage of amendments to the very defective medical practice act, and a bill legalizing dissection in our medical colleges and asylums. Dr. Wilson, the de facto chairman of that committee, will do that. In these efforts the committee had the cordial support and assistance of the county medical societies, and everything was done that could honorably be done to enlighten the legislators on the relation of sanitary science to the public health, and the necessity of putting additional restrictions upon the practice of medicine. Our arguments were in vain. We were unable to cope with the influences brought to bear by persons interested in the defeat of that bill. It is hard to understand how, without influences other than reason and argument, a handful of men representing sects in medicine could negative the influences of the combined medical profession of Texas, and it is not very flattering to realize that such has been the case.

Our anatomical bill was treated with the greatest discourtesy, nay, even contempt. It was ridiculed, outrageously, laughed at, and overwhelmingly defeated in the Senate. It was not even accorded the courtesy of a vote, but was overwhelmed with a torrent of ridicule and abuse. The Senator who so ably advocated our medical practice bill last session, and on whom we relied for support this year, disappointed us greatly; and with regard to the anatomical bill, he made a jest of it and wanted to amend it by making it applicable only to doctors. "Let them dissect each other," he said. Senators Terrell and Chambers distinguished themselves in lengthy speeches of ridicule and denunciation of those who would condemn a poor man to dissection because he was poor and friendless.

Senator Looney, who introduced the bill at the request of your committee, made a noble defense of it, and presented an argument which was unanswerable. He pointed out that the State has founded a great medical college for the education of the physicians of the future at the expense of the taxpayers, and that there are other medical colleges in the State; that annually there is appropriated something like $50,000 for the support of the medical department of our great university; that anatomy and pathology, which can be learned only on the cadaver, form the basis of medical education; that a professor of anatomy is paid $4000 salary to teach it; that the State requires a profound knowledge of these branches on the part of the student, as a condition to the doctor's degree, and an additional examination by the State board of med-

Dr. D. F. Stuart,
Houston, 1873.

Dr. S. C. Red,
Houston, 1902.

Dr. S. R. Burroughs,

Buffalo, 1887.

Dr. Frank Paschal,

San Antonio, 1903.

Dr. R. H. Harrison,

Columbus, 1876.

Dr. Bacon Saunders,

Fort Worth, 1897.

Dr. P. C. Coleman,
Colorado City, 1895.

ical examiners as a requisite to license to practice; and yet the law makes it a felony to procure the material necessary to the study.

The passage of this bill would have done more to protect the family burial ground than all the penalties attaching to grave-robbing. For there are persons who will risk the law in order to supply a commodity for which there is a cash demand, and the material for the study of anatomy must be forthcoming from some source, or we should abolish the medical school. This bill, he explained, sought to remedy that condition, and make dissection legal; and that the bodies of those who die in our charity institution, if unclaimed after notice and publication, constituted the only available material. But Senator Looney could not stand up alone, or almost alone, before such an onslaught of ridicule, abuse of the doctors, and maudlin sentiment. He deserves the thanks of our body and to be remembered. The scene was as insulting to the faculty of the State University and to this Association as it was disgraceful to those who engaged in it, and to the State of Texas, and to the enlightened age in which we live. I protest that any request coming from such a source is entitled to the respect due even from one gentleman to another, and I protest against the brutal treatment accorded to this effort to advance the cause of medical science and the public good.

I am required to submit to you recommendations "for the good of the order." Naturally, medical legislation should first be considered. But I can not reconcile it with my own self-respect nor with the respect due you to recommend another effort to regulate the practice of medicine. The State Medical Association has proceeded always upon the assumption that the lawmakers can not intelligibly legislate upon medical and sanitary matters without the counsel and assistance of the learned men of the medical profession; and in this, so far, you are right; but we assume further that they desire that assistance and advice. We have volunteered it; it has never been asked for, and it has been ignominiously rejected time and again and this time ridiculed. They are sufficient unto themselves, and we have been repeatedly told so by the rejection of everything we have ever suggested or asked for for the prevention of disease or the advancement of medical science. And as for the protection of the innocent and ignorant from the rapacity and danger of the quack, they can not be made to see it as we do. Our law is a seductive bid to the quackdom of the world to come to Texas. Here they can fake the people without let or hindrance. A bill for

A STATE BOARD OF HEALTH, to be presented to the Thirtieth Legislature will be laid before you for your action. It was carefully drawn up by your committee on public policy and legislation, and it has been approved, in the main, I think, by all the county societies. I recommend that every proper means be used to secure the passage of that bill—if you think, in light of our past and recent experiences, it is worth while. Such a measure is of the greatest importance, and it is the State's duty to enact such a law, but we have never been able to make any Legislature realize the first nor admit the latter proposition. It would "cover the ground" of sanitary science and would make unnecessary special legislation, such as regulating the sale of patent medicines, the sale of narcotics and other poisons, of adulterated foods, drinks and drugs, preventing the pollution of water supply, the disinfecting of schools, churches, hotels, cars, etc., all of which should be regulated by such a board; and the board should have the power to regulate the practice of medicine, and to be the only licensing body, as in Alabama. But in the existing state of legislative sentiment we would do as well to ask them to abolish the moon. Such hope is utopian. It can never be realized until the people of Texas are awakened to a sense of the needless loss of life by preventable diseases and the sacrifice of life by the exercise of their boasted privilege of "employing any doctor they want," licensed by the State or not. Will no argument avail to make the lawmakers realize that for every death from yellow fever in America there are 150 from consumption? And that, like typhoid fever, malaria, dysentery and many other diseases so destructive of life, its spread, at least, can be checked. I recommend that in every county a committee, or one person, be appointed to present from a broad, rational and comprehensive standpoint the cause of sanitary science in its relation to the public health by public addresses and by newspaper publication several times a year, the publications to be paid for if necessary. An intelligent member of each county society, I dare say, could be induced to give an occasional lecture or public address on the subject in the capital of his county. Much missionary work can be done by every physician in enlightening his patrons and in asking the voters of his clientele to ascertain whether or not the candidates in his county for seats in the Legislature are enlightened or if, when sent to Austin, they will laugh at those who are seeking to banish disease and put themselves out of commission as it were, or if they will be governed by reason and common sense and not by a silly sentiment and a mistaken sympathy for the bottom dog?

### SECTION ON STOMATOLOGY AND ORAL SURGERY.

Dentistry is recognized as a branch of medicine by the American Medical Association and by many State associations. There is a State association of dentists with a membership of about 200, and they have a board of dental examiners. None but graduates in dental surgery are admitted to membership. I recommend that a special comittee of five be appointed to confer with a similar committee from the State association of dentists, which meets in Austin in May, looking to a merging of their association into ours, or at least to an affiliation and admittance of properly accredited members of that organization, to membership in our Association, and the creation of a section on stomatology and oral surgery.

### AMENDMENTS TO CONSTITUTION.

Our constitution and by-laws need amending in several particulars:

The State Board of Medical Examiners has always made a report to the State Medical Association ever since the passage of the law establishing that body. They appear to be a kind of regular standing committee without the constitutional standing. The law requires that they shall have practiced medicine for not less than five years, having complied with the laws relating to the practice of medicine in force in Texas, for not less than five years prior to their appointment. They shall be appointed by the Governor on the 10th day of May following his inauguration from a list of names twice the number to be appointed, to be furnished him by the Texas State Medical Association. This board should be recommended at this meeting. I would suggest that there be inserted in the by-laws a clause directing the appointment of eighteen members at the proper time required by law, and that the board appointed by the Governor be constituted a regular standing committee of the Association; such committee to be published in the transactions under the head of regular standing committees, and that an annual report before the House of Delegates be required of this body.

### BOARD OF TRUSTEES.

Our articles of incorporation, which make us a legal corporate body, section 6, state that "its board of directors or trustees shall consist of five members," and the names and places of residence of those to serve the first year, respectively, are as follows: B. E. Hadra (deceased), Dallas; Taylor Hudson, Belton; R. F. Miller, Sherman; H. P. Cooke, Galveston; H. A. West (deceased), Galveston.

We are a corporate body, and as such have a legal status before the world to sue and be sued, and to own property and conduct business like other corporations. The original board of trustees (no others have been elected) still continue as the legal representatives of the Association until their successors are elected or appointed. It is not possible for us to maintain our charter and not comply with the requirements. Our constitution and by-laws do not provide for the election of trustees, nor define their duties.

### DISTRICT SOCIETIES.

Your attention is called to article 6 of the constitution. It provides "for the organization of such councillor district societies as will promote the best interests of the profession." Several district societies have been created, but their status in relation to this body and their duties and powers and limitations are not defined. This matter requires your serious consideration. Our organization is not complete without it.

### THE TRANSACTIONS.

I recommend, as every president for twenty odd years has done, to continue the publication of the transactions in book form, uniform with the volumes since 1884. I do not believe that a change to a journal would be for the best interests of the Association or would meet the approval of the members who care for and preserve these handsome and valuable volumes in their libraries. Nor do I believe that our finances would enable us to establish a journal large enough to contain the matter which now makes some 700 pages, after cutting out certain papers and giving offense to their authors. It will be remembered by the older members that the experiment was tried and proved unsuccessful, and the volume form was resumed in 1884. Thus a gap or hiatus in our history was created, which can never be bridged over—the journals are lost.

### THE PROCEEDINGS.

The President's address and recommendations were referred to a committee of three to be appointed by the chair, to be subdivided

Dr. H. C. Ghent,

Belton, 1884.

Dr. T. Hudson,

Belton, 1901.

Dr. S. C. Red,

Chairman Entertainment Committee.

and submitted to such other committees as the subject-matter may require, whether standing or special.

Reports from all the councillor districts, showing a creditable condition of affairs, from an association standpoint, were received and filed.

A verbal report from the committee on scientific work was received, and further time was given other standing committees.

HOUSE OF DELEGATES.

After a few councillor reports had been submitted, Dr. Burroughs reported for the committee on recommendations in the President's message. On the matters of medical legislation, it was recommended that the matters be referred to the committee on public policy and legislation. As to the proposed constitutional amendments, it was suggested that a standing committee on constitution be appointed to take up the matters along such lines. The scientific divisions indicated by Dr. Daniel were ordered referred to the committee on scientific work. The matter of affiliation with the State Dental Association, it was suggested that the chair appoint a committee to take up the question. Suggestions regarding district societies were referred to the trustees. The report of the committee was adopted.

Dr. B. F. Kingsley was named by the president as chairman of the committee on constitution, and Dr. A. Garwood was named as chairman on conference with the Dental association. These are to name the remaining members of the committees.

For the comittee to select a permanent place of meeting for the society, Dr. McLaughlin stated that the committee had concluded that the time had not yet come for such action on the part of that body. Report adopted and committee discharged.

The committee on conference with the Pharmaceutical Association reported nothing accomplished.

The committee on institution for indigent consumptives filed a splendid report. It recommended the purchase of the old army post buildings at Fort Davis as the cheapest step to be taken with satisfactory results. The committee was continued in the hope of securing State assistance.

ANNUAL REPORT, BOARD OF MEDICAL EXAMINERS, DR. J. T. WILSON.

Pursuant to custom the Board of Medical Examiners for the State of Texas presents for your information the following report of its transactions for the year, with a brief resume of its work from the beginning:

There have been two regular meetings since the last report. The usual spring meeting was appointed for the 2d day of May instead of in April, as formerly. The first two weeks in April being inconvenient for a majority of candidates, and the third week being too near the meeting of this Association for the board to finish its work and have its report ready, and as the terms of the members expire on the 10th of May, it gives the board a better opportunity to have its affairs in proper shape at that date. Because of the spring meeting having been deferred until May it will curtail this report about one-half. It will be necessary for the new board to hold a regular meeting in June for its organization, for the benefit of the graduates of the State University and of those schools whose terms end the latter part of May. There have been two regular meetings of the board since the last report. One at Houston in June and one at Dallas in October. At the meeting at Houston there were sixty-five (65) applicants. Two (2) females and sixty-three (63) males. Sixty-three (63) whites and two (2) negroes. One came in late, began his examinations, but thinking he could not complete or pass them, withdrew. Of the sixty-five (65) applicants forty-six (46) passed successful examinations and were licensed. Nineteen (19) failed. Of the failures eighteen (18) were white males and one (1) negro. The two females and one negro man passed.

At the October meeting in Dallas there were present thirty (30) applicants, all white males. Of this number nineteen (19) passed successful examinations and were licensed. Eleven (11) failed. One, when about half through with his examinations, appeared in the hall in a state of thorough intoxication, was removed and expelled. This was the first instance of the kind that had occurred in the history of the board and was greatly regretted. This young man, after he became duly sober and a week or two had elapsed, seemed much humiliated, and in a straightforward, manly way made a personal apology.

[The following is a brief statement of examinations made at the two regular meetings of the board, licenses issued and other items of interest for the year to this date: Omitted.]

There have been several prosecutions and convictions coming to our knowledge. The constitutionality of the law has been attacked, and is now threatened by the physio-medicists. We know of only one instance in which it was held to be unconstitutional, that by a lower court at Weatherford. The Court of Appeals, in an opinion handed down a short time ago, declared it to be constitutional.

The board is compelled to report the defeat, in the Legislature, of the bill embracing the amendments to the medical law as it was adopted by this house.

The clause recommended to be stricken out, which exempts the so-called drugless people, was the cause of its defeat. A new bill was drafted by the attorneys of this Association and by committees in both Senate and House, none of which we are sure, could have been indorsed by this Association, but fortunately there was not much prospect of any of them becoming a law. The history of this legislation has been submitted in detail by the legislative committee, and it will, therefore, not be necessary to allude to it further here, except to state that an amendment to this law by which the irregulars are sought to be in any way controlled will be most difficult to pass, if it ever can be. It will be opposed to the bitter end, and it seems, defeat all other amendments that would be otherwise unobjectionable to legislators, but of paramount importance to the bill. The attempt to pass laws for the creation of other boards, would seem, is an attempt to evade the law, to open the door for the admission of every character of fraud and quack. For these people to have boards of examiners composed of members of their own sect exclusively, we can not help but feel that the examinations would be a farce, possibly licensing any one who could afford to pay the fees, because we have good reason to believe they are not educated in the fundamental branches of medicine. For the State to recognize every sect who organize themselves in a distinct body and announce an exclusive dogma, legalize them by giving them a board of examiners and a law to protect them, legislation would be farcical and the State would soon be overrun with every character of charlatan. Such recognition impedes the progress of medical science. Any one who came into the State with any sort of a dogma which it is proposed to practice, would have a right to demand special legislative protection under which to ply his trade. It occurs to us that because of this tendency to protect the various isms and dogmas by the States it is all the more important that the regular profession should put forth greater efforts to elevate its standard of requirements, that its schools should be more particular in the selection of its students, demand better men and better prepared men for the complex study of medicine, that the course should be lengthened, the equipment improved, the facilities for study extended and in consequence its graduates would be more scientific practitioners, the better able to cope with the ignorant and susceptible and more able defenders of scientific medicine.

Then there is an important missionary work for the regular profession among the laity, to teach them the great value of hygiene and the great necessity for a more enlightened knowledge among them in regard to real science as applied to the principles of medicine, and thus we may expect and hope to break down the barriers of superstition and the strange fascination for things that seem to be involved in mystery, all of which are such potent factors in preventing just legislation.

The board desires to tender its thanks to the Association for its support and beg a continuation of the same.

### RAILWAY SURGERY.

A lengthy program was disposed of in general session. The section on railway surgery was at work all day under the direction of its chairman, Dr. C. A. Smith, of Texarkana. All of the papers read in this section bore more or less upon the subject of injuries received upon railroads, and covered all the ground from the organization of the medical department of a transportation company to the giving of testimony in railroad damage suits. The medicolegal aspects of railway injuries were discussed and the "invisible railway injury," as well as the so-called railway spine, were analyzed with no gentle touch. Allusion was also made to the ruses employed by lawyers to confuse witnesses on the stand with a view to casting doubt upon the reliability of their testimony, one member going so far as to say that he would call any lawyer to personal account who might undertake to go beyond the legitimate in attempts to break down his testimony—and he looked and talked as if he meant it, too.

### CARE OF THE INSANE.

During the afternoon the section on psychology and medical jurisprudence held a most interesting session. The chairman of this section is Dr. John S. Turner, superintendent of the State asylum for the insane at Terrell. His address, which was upon the subject of the care of insane patients in all stages, was exhaustive and contained many valuable suggestions regarding the treatment patients should receive at the beginning of mental trouble, his theory being that proper care at the beginning of the

Dr. J. C. Loggins,
Ennis,
President 1896.

Dr. Frank Paschal,

San Antonio,

President 1903.

Dr. R. F. Miller,
Sherman,
Treasurer.

DR. C. E. CANTRELL,

Greenville,

Vice-President.

DR. S. C. RED,

Houston, President 1902,

Chairman Entertainment Committee

DR. JOHN T. MOORE,

Galveston,

Vice-President.

development of insanity will often prevent an aggravated or permanent attack. Among other things he criticised the prevailing method of dragging the insane into court houses for trial with their subsequent incarceration in filthy jails in the company of criminals for detention until room could be found for them in the asylum.

Dr. J. T. Searcy, of Tuscaloosa, Ala., for a number of years superintendent of the insane asylum of his State, and Dr. John Punton, a noted specialist of Kansas City, followed Dr. Turner with papers on special subjects, and Dr. J. H. Eastland, of the Texas Institute for Epileptics at Abilene, read a treatise upon the care of epileptics. Dr. H. C. Ghent and others completed the program with specially and ably prepared papers. The discussion of the different papers submitted brought forth a fund of expert knowledge, Dr. Graves, superintendent of the State asylum for the insane at San Antonio, being among those taking part therein. The following distinguished guests sat with the section during its session: Dr. A. E. Macdonald, emeritus professor of nervous diseases in the Bellevue Hospital School of Medicine of New York; Dr. Dyer, the eminent cancer and skin affection specialist of New Orleans, and Dr. Van Wort, a noted neurologist of the same city.

The committee on constitution and by-laws reported a provision giving the board of trustees a constitutional standing and recommended an amendment to the by-laws providing for a three instead of a four days' annual meeting. The report was concurred in and filed.

A report submitted by the board of councillors was adopted. It provided for the transfer of Erath and Johnson counties from the Fourteenth district to the Twelfth and the removal of Freestone county from the Twelfth to the Eleventh district and the transfer of Mills and Lampasas counties from the Twelfth to the Fourth district.

The committee on the President's message, for the section relative to the formation of district societies, reported that it was advisable in some districts to form them, though not in all, and recommended that the councillors be permitted to charter such, and that they have the power to combine district societies where it was desirable to do so. The report was adopted.

Dr. J. D. Law's oration was "The Physician and the Humanities." It was truly eloquent and gracefully delivered. He declined to give out his manuscript to the *Post*.

### A MONTHLY JOURNAL.

The House of Delegates had a session during the morning at which the report of the committee on publication was received and filed. This document contained an emphatic indorsement of the proposition to undertake the publication of a monthly journal to take the place of the annual volume, giving space to the minutes of the Association and the papers read thereat. Such a publication has been the dream of many members for years, and the committee studied the subject closely with a view of determining whether the Association is in a position to undertake it. The result of this study was the submission of a mass of figures sustaining the proposition, accompanied by a recommendation that it be adopted. The report having been filed, Dr. Holman Taylor introduced a resolution committing the Association to the immediate adoption of the journal idea, the details of its carrying out to be arranged by the board of trustees. This was opposed by the President, Dr. Daniel, who, while not inimical to the ultimate publication of a monthly journal, was not sure that the time was opportune for inaugurating the change. He wanted a further study of the matter and a more thorough investigation of the item of expense incidental to the proposed undertaking. Vice President Moore, Secretary Chase, Treasurer Miller, and Dr. J. T. Wilson spoke earnestly in support of Dr. Taylor's motion. They were satisfied with the figures submitted and felt confident of the Association's ability to carry the project through. Furthermore, they had no doubt but what a monthly journal would prove a powerful agent in the upbuilding of the Association and stimulate interest in its work.

Dr. Taylor's resolution was adopted by a unanimous vote.

### MEMORIAL SERVICES.

At the conclusion of the lecture memorial exercises were conducted by Dr. I. C. Chase, who took charge of them in lieu of Dr. J. A. McGee, who was prevented from attending this meeting by sickness.

The initial number on the program was given by the Apollo quartette, which sang "Nearer, My God, to Thee." The quartette is composed of Duncan Bell, first tenor; J. L. Carr, second tenor; George B. Meyer, first bass; Herbert R. Gates, second bass; H. C. Breaker, director.

The quartette having concluded, Dr. Chase read eulogies upon the departed members of the Association, who have died during the

DR. J. W. SCOTT,

Houston,

Chairman Reception Committee.

DR. R. W. KNOX,

Houston,

Committee of Arrangements.

DR. F. E. DANIEL,

Austin,

Pres. State Medical Association

Dr. Sam R. Burroughs,

Buffalo,

President 1877.

Dr. J. D. Osborn,

Cleburne,

President 1892.

Dr. Taylor Hudson,

Belton,

President 1901.

past two years, which were prefaced by Dr. McGee, each eulogy being accompanied by a presenting of the countenance of the deceased by means of a stereopticon.

The thus remembered dead were Drs. B. E. Hadra, N. H. Dixon, H. A. West, E. R. Manning, John C. Jones, V. C. Lunn, John L. Hall, Henry C. Roberts, Judson Wade Garnet, J. T. McDonald, M. V. B. Thornton, W. B. Pullen, J. B. Moeur, J. F. Ford, William Pannell, A. S. Wolff, R. E. Moody, James Orr, J. D. Bass, A. C. Williamson, J. F. Moore, Sr., E. R. Hawkins, Henry Hart, C. E. White, H. J. Chapman, F. B. Clarke, Jack Phillips, W. Y. Winton, E. J. Melish and G. S. West.

Dr. R. F. Miller read a eulogy upon Dr. Joshua Larendon and Dr. Daniel paid feeling tribute to the memory of Dr. George S. West, who died recently at the age of 83 which was most expressively responded to by the entire membership.

The quartette sang Tennyson's "Crossing the Bar," and the services were brought to a close by a motion to adjourn, which was put and carried.

### OFFICERS ELECTED.

President—Dr. J. E. Gilcreest, of Gainesville.

Vice Presidents—Dr. M. B. Grace, of Seguin; Dr. Thomas A. Rape, of Ballinger; Dr. O. I. Holbert, of Waco.

Board of Trustees—Dr. W. R. Thompson (five years), Fort Worth; Dr. J. S. Lankford (four years), San Antonio; Dr. C. E. Cantrell (three years), Greenville; Dr. W. B. Blailock (two years), Dallas; Dr. S. C. Red (one year), Houston.

Councilors—Dr. L. A. Gizzard (Second district), Abilene; Dr. T. J. Bennett (Seventh district), Austin; Dr. Green Davidson (Eighth district), Wharton; Dr. John T. Moore (Ninth district), Galveston; Dr. B. F. Calhoun (Tenth district), Beaumont.

Alternate Delegates—Dr. W. B. Russ, San Antonio; Dr. W. C. Jones, Walnut Springs; Dr. W. L. Brown, El Paso.

Fort Worth was chosen as the next place of meeting, last week in April, 1906.

Dr. M. H. Moody, of Greenville, was elected orator for next meeting.

When the new president appeared he was roundly applauded and was eloquently presented by Dr. Daniel. His address was very brief. He said: "Gentlemen, this is an honor you have conferred upon me that any man in the State would be proud of. I have been a member of the Association for twenty-one years, and I love it. I have tried to discharge every duty I owed the Association in the past and I assure you I will try to serve you in the best way I possibly can as your president and shall spare nothing toward the upbuilding of this organization. I appreciate the responsibility imposed upon me. The Association has experienced a remarkable growth in the past three years; I trust it will continue to go forward, and it shall be my endeavor to keep down friction and have all move forward smoothly. I feel that you could have found others more capable, but I will do the best I possibly can. I sincerely thank you for the honor."

Each of the three vice presidents were presented by President Daniel, and they made brief but delightful little talks which were heartily received.

Dr. M. H. Moody, the 1905 orator, was introduced, and though he spoke but a few minutes, he convinced the delegates that no mistake had been made in selecting him to represent the Association on the platform next year.

On retiring from the office and relinquishing the gavel, Dr. Daniel made an eloquent talk. He spoke especially regarding the failure of the State Legislature to take up needed reforms in laws governing medicine and stated that though he had labored long and faithfully he had not reaped the success he had anticipated and hoped for.

Dr. Daniel was given a rising vote of thanks by the delegates.

A rising vote of thanks was tendered Secretary I. C. Chase and Treasurer R. F. Miller, both popular officials of the Association. These gentlemen yet have two years to serve, they being elected last year for a term of three years. It has been the common expression of the members of the Association that Dr. Chase has been a power of good in the office of secretary, the duties of which he has discharged so successfully.

A vote of thanks was extended Dr. E. B. Blalock, of Harrison county, Dr. J. S. Miller, of Milam county, and Senator Grinnan, members of the State Legislature, for services rendered the Association in legislative matters.

### REPORT OF COMMITTEE ON PUBLIC POLICY AND LEGISLATION.

Chairman J. T. Wilson, of Sherman, submitted the report of the committee on public policy and legislation, which was an exhaustive review of the work done and the failure of the Legislature to properly consider the demand for proper laws on medicine and medical schools.

REPORT OF TREASURER.

Dr. B. F. Miller, of Sherman, treasurer of the Association, submitted a report which shows the society in a splendid condition. The balance from the April, 1904, report was $4795.57. The total receipts to April 25, 1905, were $10,439.16. The total expenses for the past year were $5343.97, leaving a balance on hand of $5095.19.

Dr. J. F. Y. Paine, Galveston, President 1888.

Dr. J. W. McLaughlin, Galveston, President 1894.

SECRETARY'S REPORT (IN PART).

Secretary Chase submitted his report and the same has been adopted by the House of Delegates. In part it is as follows:

Your secretary submits the following:

At the April meeting, 1904, the secretary reported 2415 members. It was quite impossible at that time to have complete reports from all counties. After the meeting it appeared that 35 members had paid twice and a considerable number of societies had reported membership for which they could not collect. After accounts had been balanced the number of members actually paid for at the last meeting was 2263. During the year 130 paid members were secured before the publication of the transactions. These were added, making 2393 reported in the volume.

Texas ranks seventh in the list of States in the number of its physicians (4826), fifth in membership of State societies (2393), twelfth in percentage of membership to total number of physicians, last year enrolling 49.5 per cent of its practitioners, or practically one-half. Excepting Massachusetts, no other State society of its size enrolled so large a percentage of its members. There are more doctors in Texas than in any other Southern State. Tennessee stands next with 3428, Kentucky follows with 3372, and Georgia with 2780. Texas has more members in its State society than any other Southern State. Excepting Kentucky, which has about 1361 members, and Alabama, 1295 members, Texas has twice the membership of any other Southern State.

The present membership is 2436, showing an increase over last year of 43 members (173 more than last April).

Seven new counties have been added since the publication of transactions, with a membership of 86. Tyler county has failed to renew. Mason and Llano counties have been united. In addition to this, Orange has only renewed 2 and Burleson 2.

The labor involved in collection and transcription is immense. During the year 2167 complete professional records were secured from members and 180 complete records from non-members, in all 2347. The correspondence necessary to complete the data for this work in some measure explains the large postage account. The legislative campaign likewise increased the postage and telegraph accounts. There is still a great work to be done toward securing the records of the remaining 2479 physicians in Texas. It will be very difficult, as county secretaries are not active in securing professional records of non-members. So far as I know, only one county, Smith county, has completed the professional canvass of the county under the untiring leadership of Dr. Albert Woldert, of Tyler.

Collections for the year, ending at the Austin meeting, were $5986. The total number of actual paid members proved to be 2263. These members should have yielded an income of $4526. This indicates that $1460 were actually collected more than regular dues, and were obtained, I believe, from delinquent members on the $5 plan and from numerous members who paid $5 in the place of

$2 to the State. This was a fortunate occurrence for the Association, but such financiering can not be repeated.

Collections for the year are $5643.59, which is three hundred and forty-two dollars and forty-one cents less than last year; $5986 have this year been received for membership, and this sum has been swelled by $472.52 received on special legislative fund and for the sale of indices and transactions. The sale of indices is not an asset, as we buy these and sell them to county secretaries without a profit.

The expenses of the secretary's office has been kept as low as the business interests of the Association would allow. We possessed no typewriter. A "built-over" Remington was purchased at $40 instead of a $100 new one. A convenient desk had to be provided. The Association has gotten along without other labor-saving devices. Outside of circular letters, packages of supplies, etc., the postage accounts indicates the writing of over 4000 letters. It will be necessary in the future to purchase a good filing cabinet, as the time required in searching for data to answer twenty letters a day is considerable.

One of the greatest difficulties of the year has been in securing competent help at the small salary available. Four different stenographers have been tried, but for the sum paid only inexperienced and immature help can be employed, requiring constant oversight and direction.

The committee on scientific work will make no formal report, save to refer you to the program as evidence of their activity, and here call your attention to our four, and for delegates five-day session. Your committee has not noticed another State society meeting of more than three days. On account of the size of the State and time necessarily spent in travel, it seems desirable that the program should hereafter be limited to three days.

HOME FOR CONSUMPTIVES.

The report of the committee on a State institution for indigent consumptives was read by Dr. M. M. Smith. Said report was in effect that, although Representatives Wilmeth and James, in the lower House, and Senator Hicks, in the upper House, had introduced bills providing for the creation of such an institution, nothing had come of them. The committee was not discouraged, however, as it hardly expected to get an appropriation at this session, owing to the depleted State treasury. Dr. Smith said that the question of providing for an institution of the character referred to was all important, as the ratio of deaths from consumption was increasing at a rapid rate, and that indigent consumptives should be isolated and cared for, the danger of infection from such persons being great. He suggested also that correspondence be entered into with the owners of the Fort Davis property in Western Texas, which could be purchased at a reasonable rate, and which would afford an ideal location for a State institution for the care of persons suffering with tuberculosis. The report of the committee was adopted and the committee continued.

Prominent among the visitors were Dr. A. E. Macdonald, emeritus professor of mental diseases in Bellevue Hospital College, New York, a celebrated criminologist and medico-legal expert; Dr. J. T. Searcy, superintendent of the Alabama State lunatic asylum at Tuscaloosa, and a noted psychologist; Dr. John Punton, of Kansas City, who is a specialist of national reputation in mental diseases; Dr. Dyer, the famous authority on cancer, who was formerly a resident of Galveston, but now lives in New Orleans. Dr. Dyer read a paper on leprosy. Dr. W. A. Van Wart, of New Orleans, was also a guest. The inimitable Dr. Emory Lanphear, of St. Louis, was present and read a paper on the "Encroachment of the Surgeon on the Field of the General Practitioner," or something like that. All the distinguished visitors were accorded the usual privileges and courtesies.

There was an unusually large and interesting exhibit of surgical instruments and appliances, books, and pharmaceutical products. But many exhibits were notably absent, because of the charge for floor space, which, I think, is a mistake. These exhibits are educational and interesting. Professor Keiller, of the medical department of the University of Texas, is an artist, as well as a most accomplished anatomist. He presented fifty wax models of his dissections of various regions.

There were twenty-three papers in the section on Railroad Surgery. The section on Surgery and that on Psychology and Jurisprudence were especially strong. The attendance was near the 500 mark. All in all it was a most harmonious, successful and satisfactory meeting. I regret that I have not room for a report on section work.

The social features of the meeting were most enjoyable. Dr. and Mrs. Red "received," and the callers enjoyed a delightful visit. Mrs. J. W. Scott gave a drive over the city to the visiting ladies, followed by a delightful luncheon. And the ball on Thursday evening, after the President's address and the annual oration, was "swell for sure enough." "Words fail," etc.

## Figures and Tables

**Figure f1:**
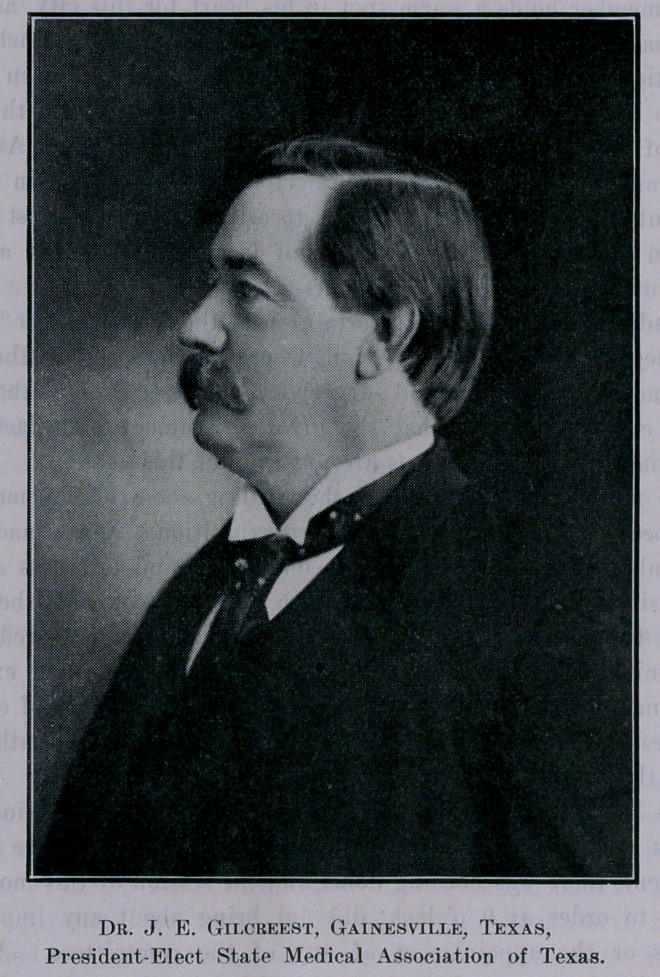


**Figure f2:**
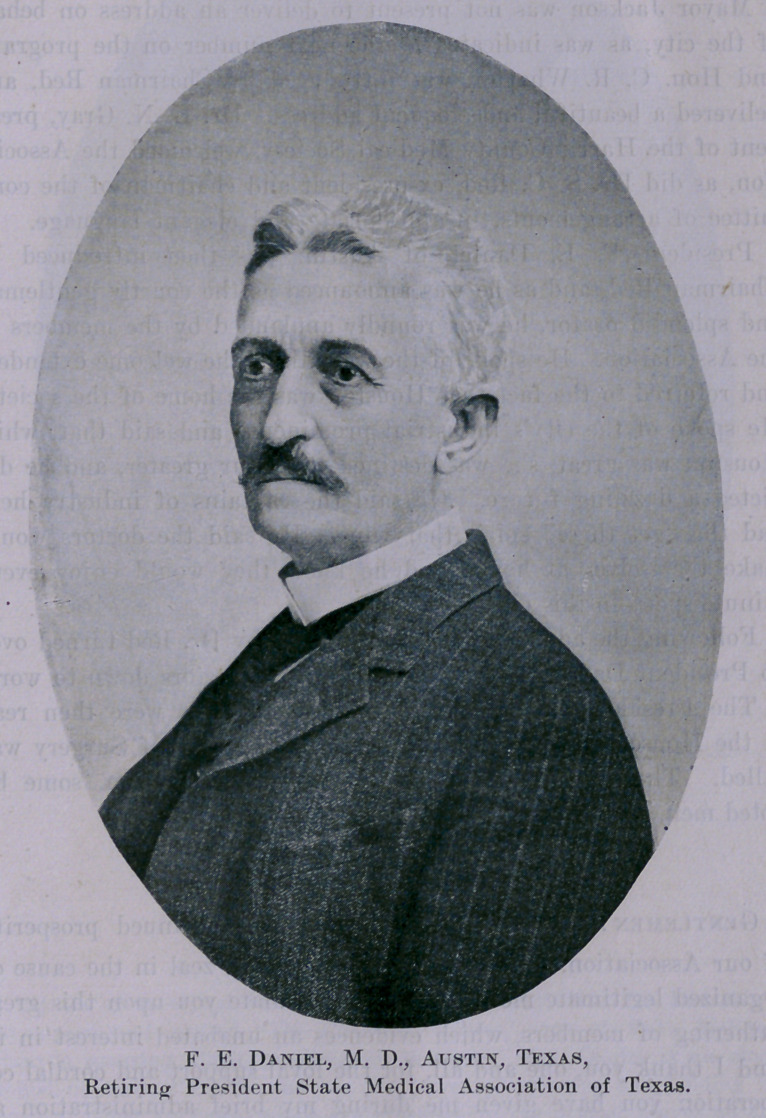


**Figure f3:**
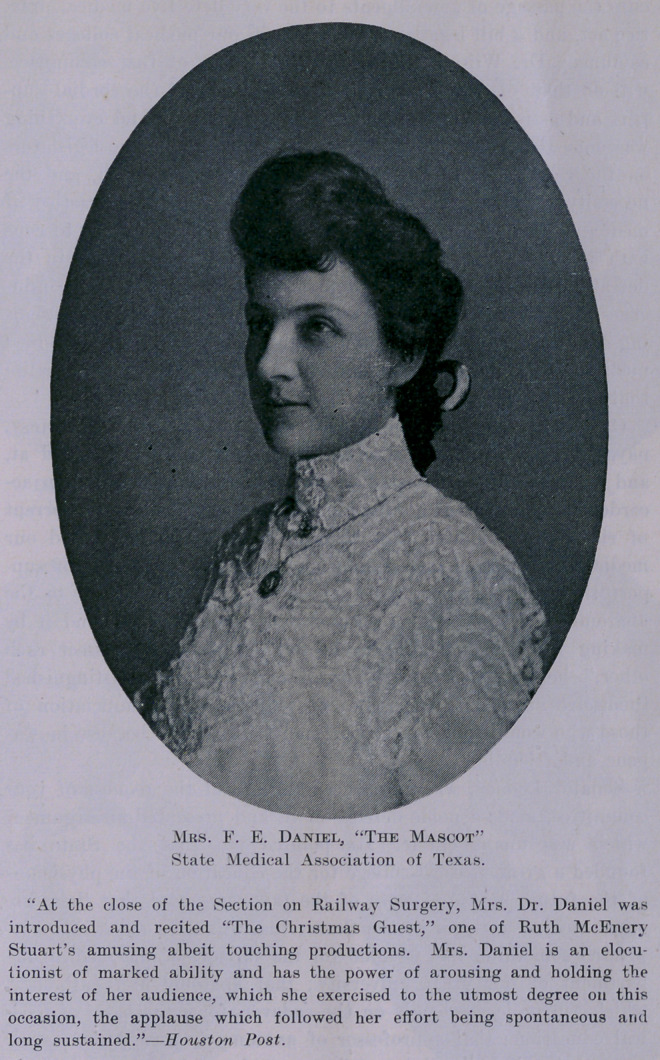


**Figure f4:**
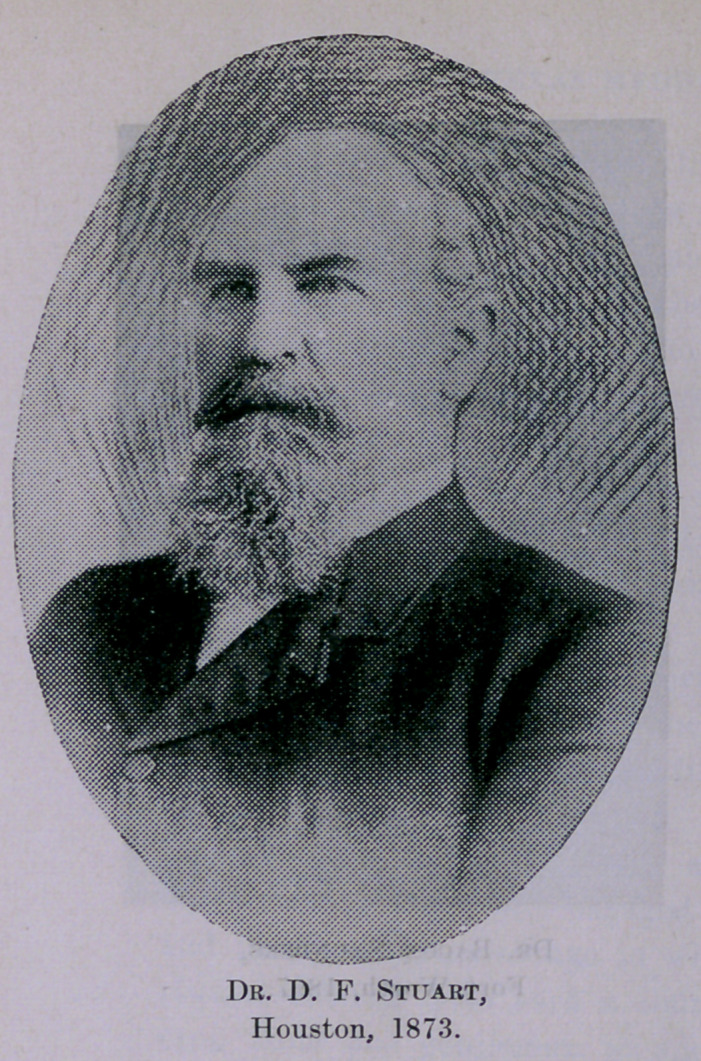


**Figure f5:**
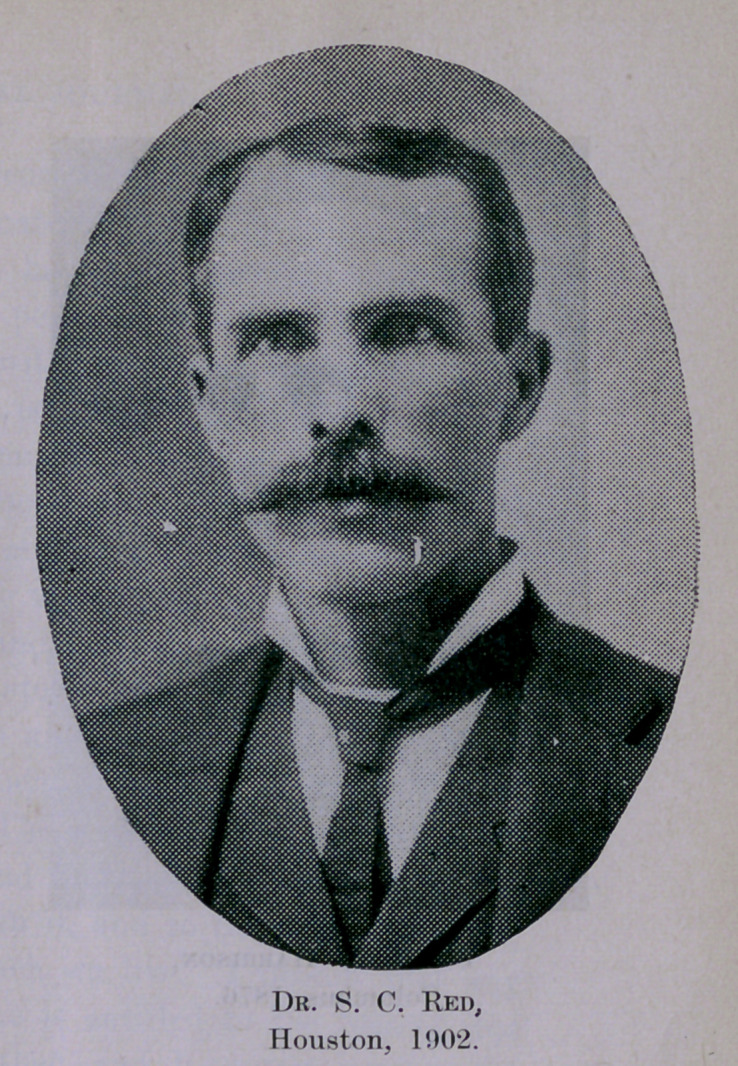


**Figure f6:**
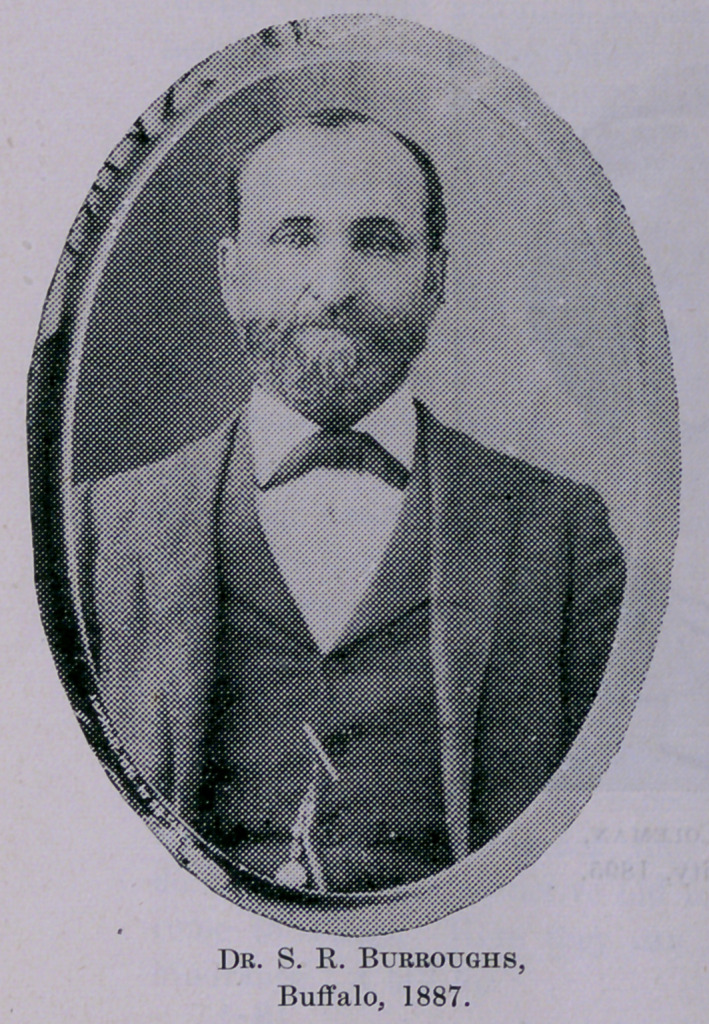


**Figure f7:**
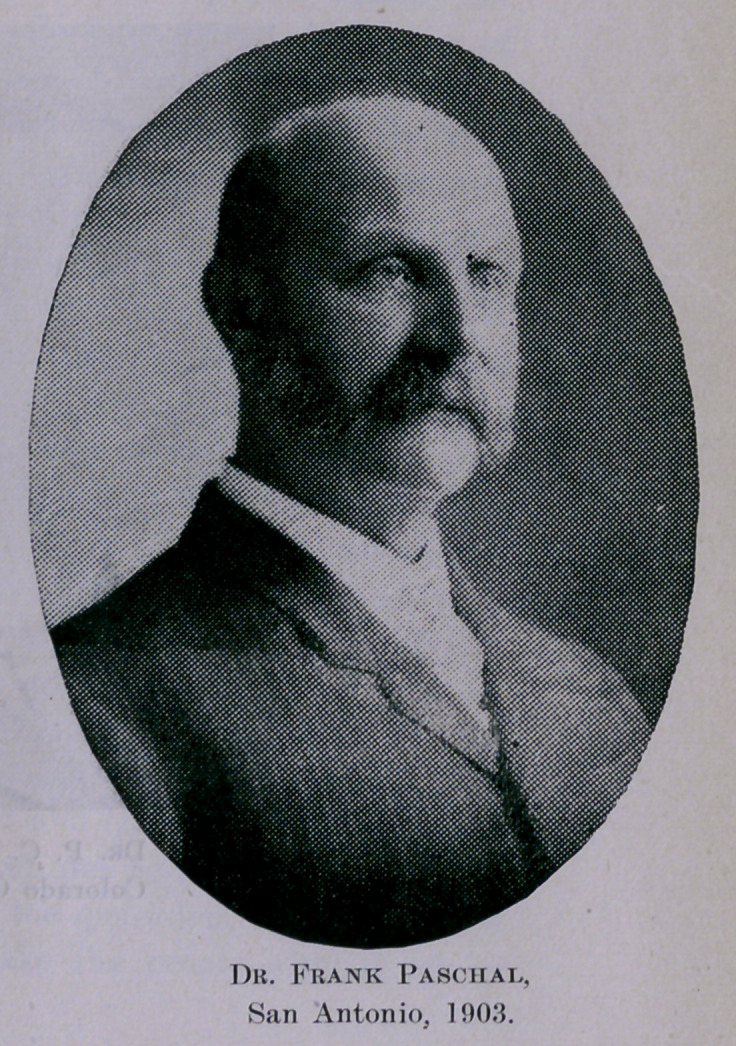


**Figure f8:**
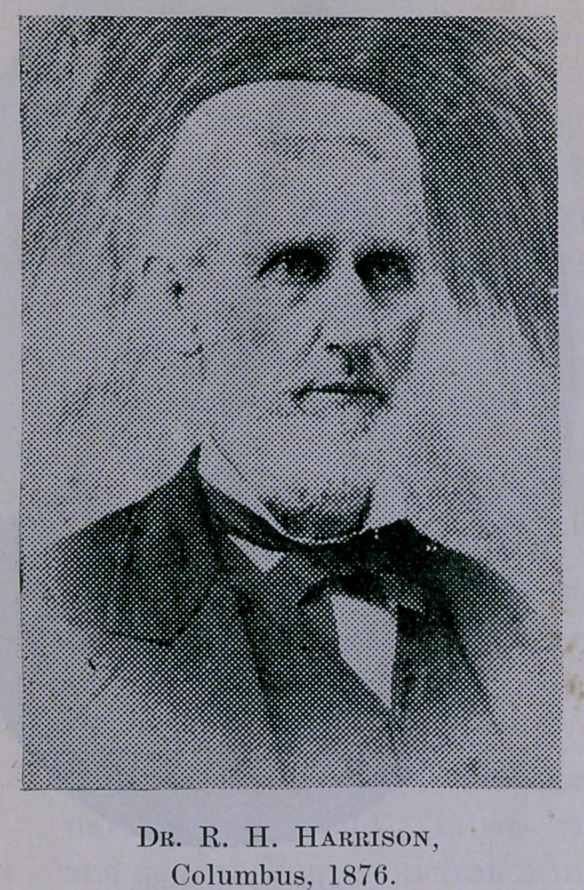


**Figure f9:**
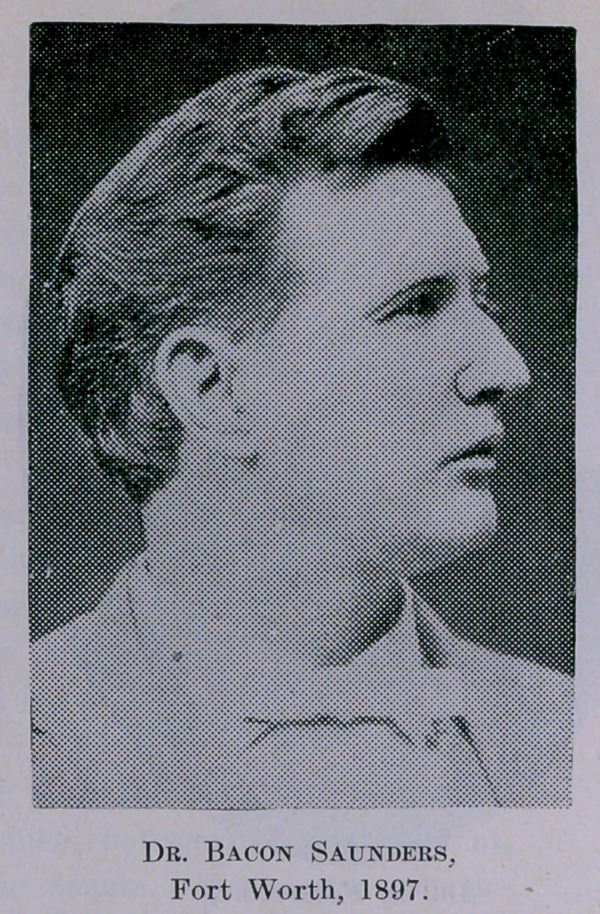


**Figure f10:**
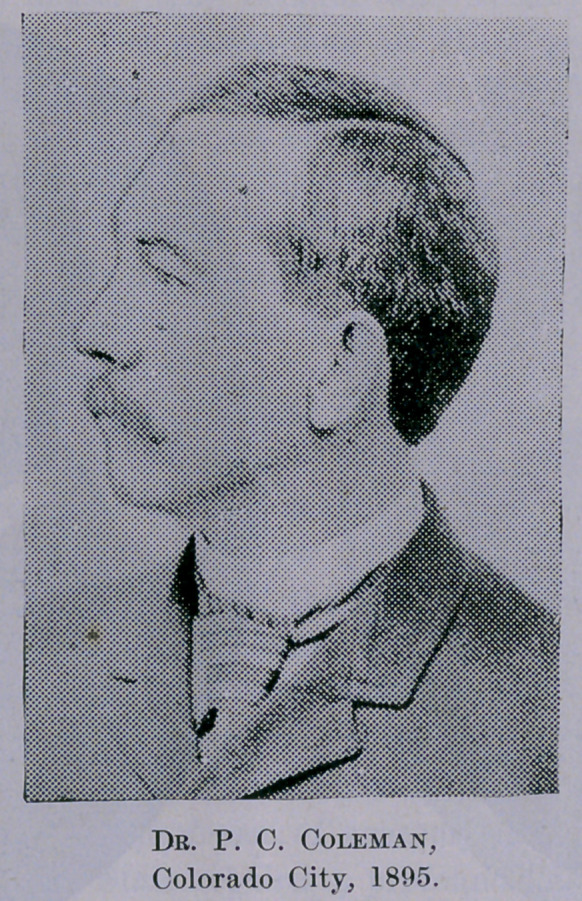


**Figure f11:**
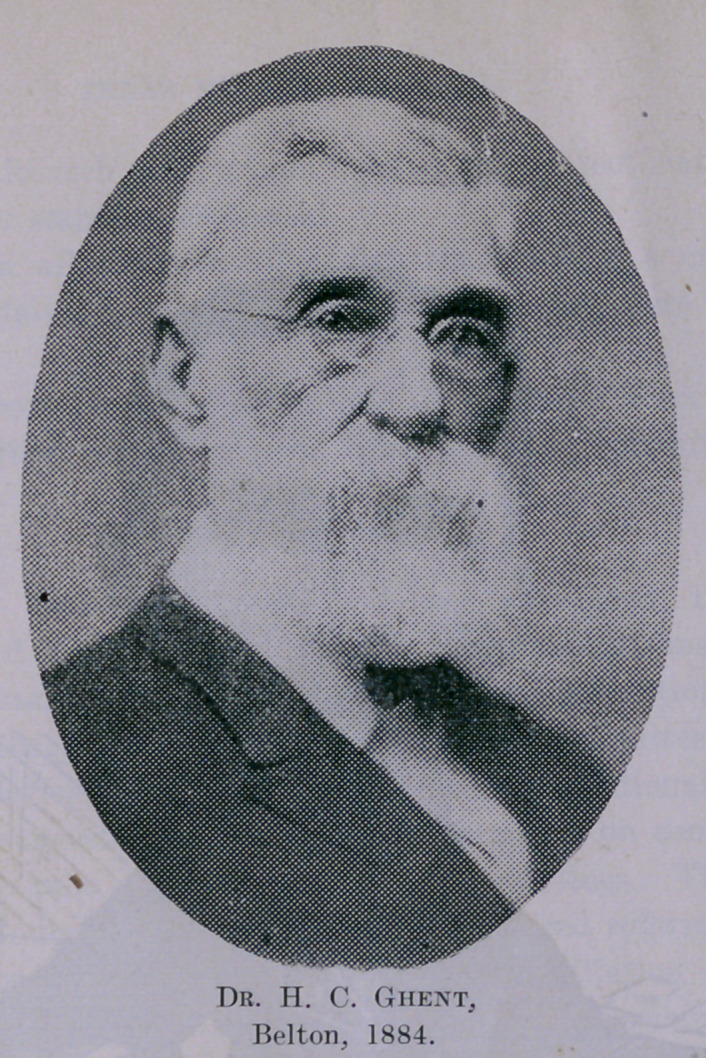


**Figure f12:**
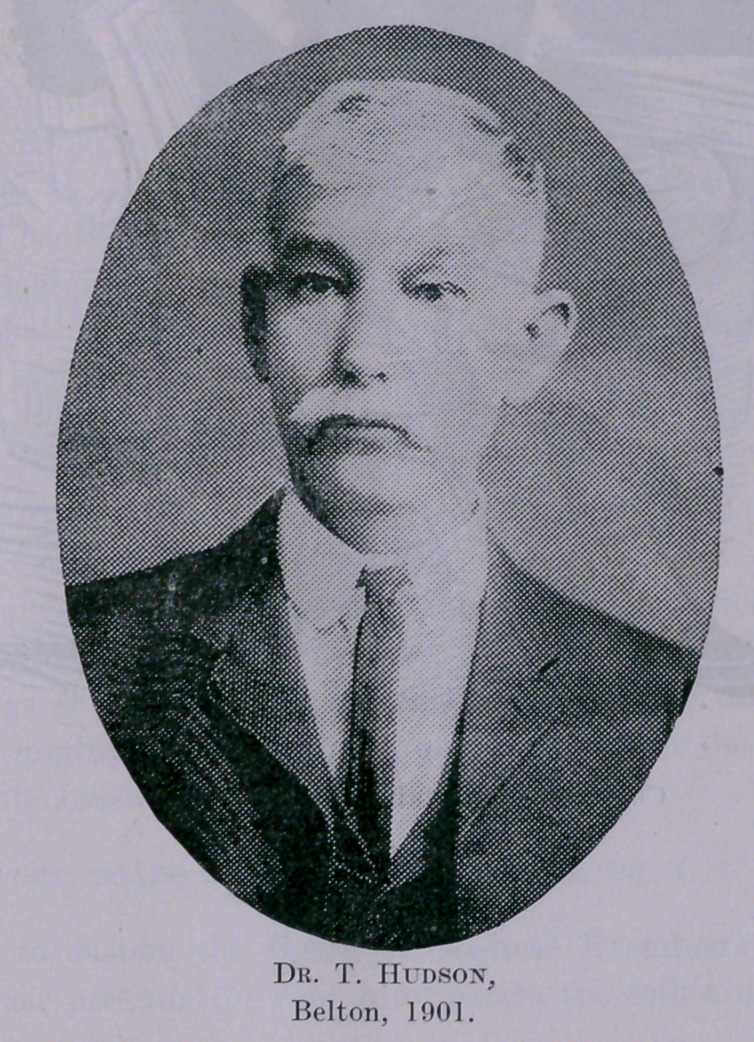


**Figure f13:**
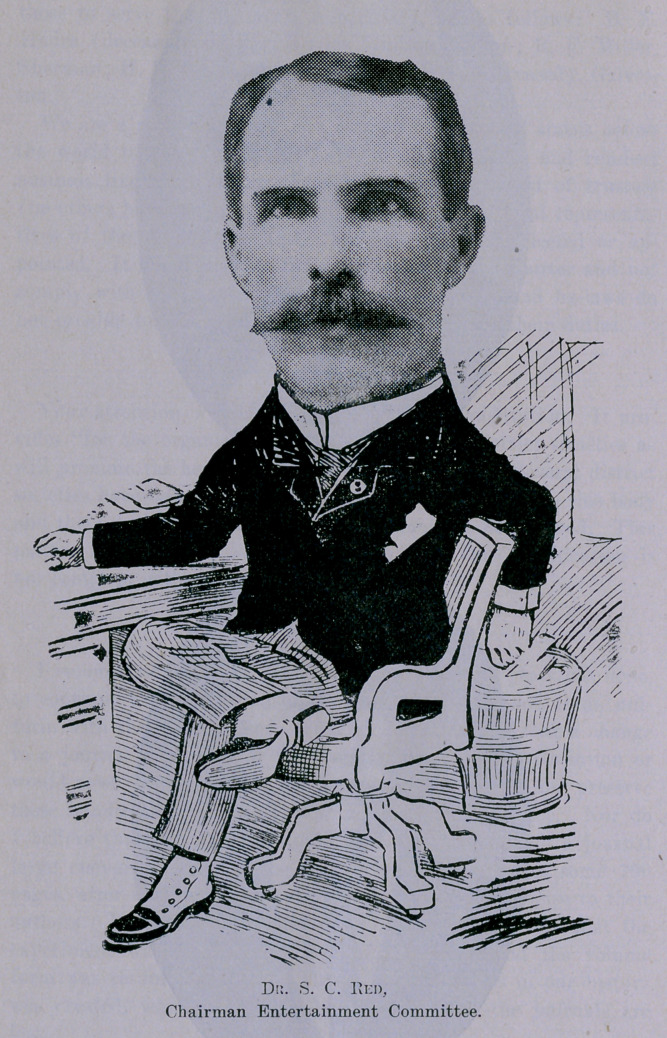


**Figure f14:**
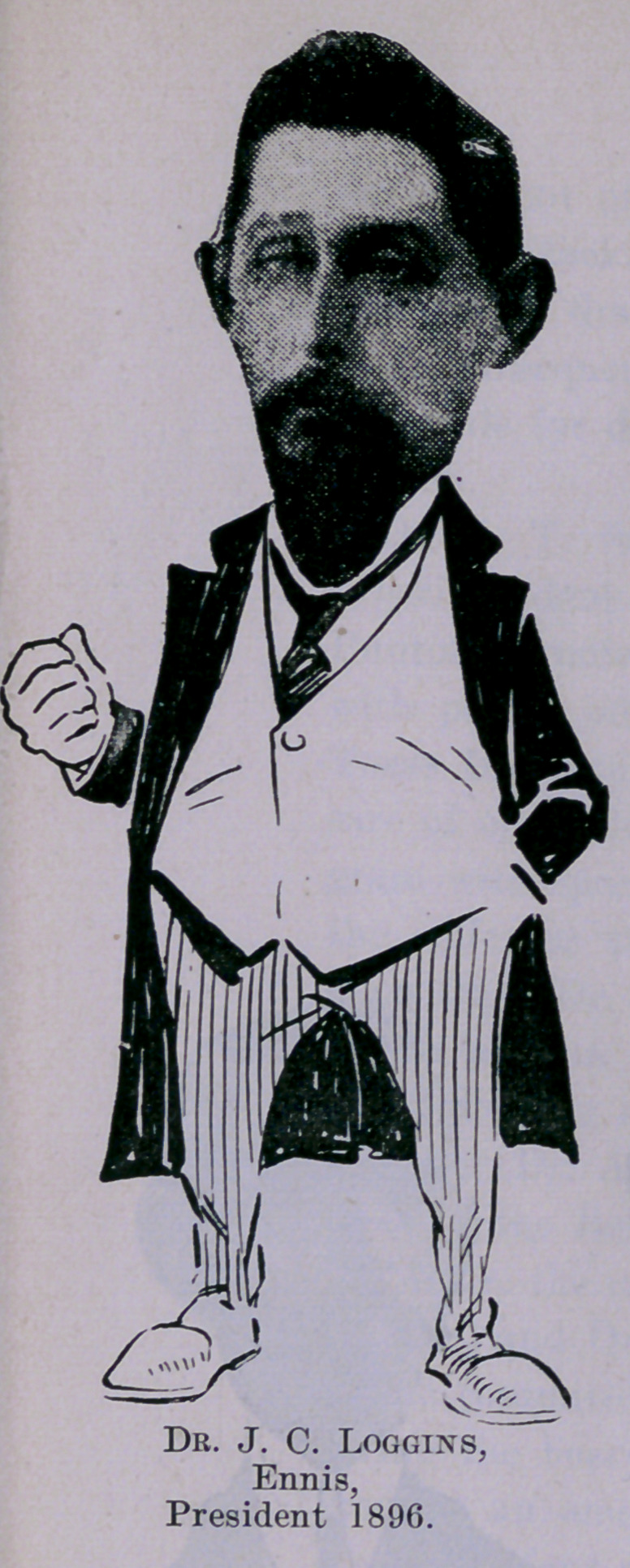


**Figure f15:**
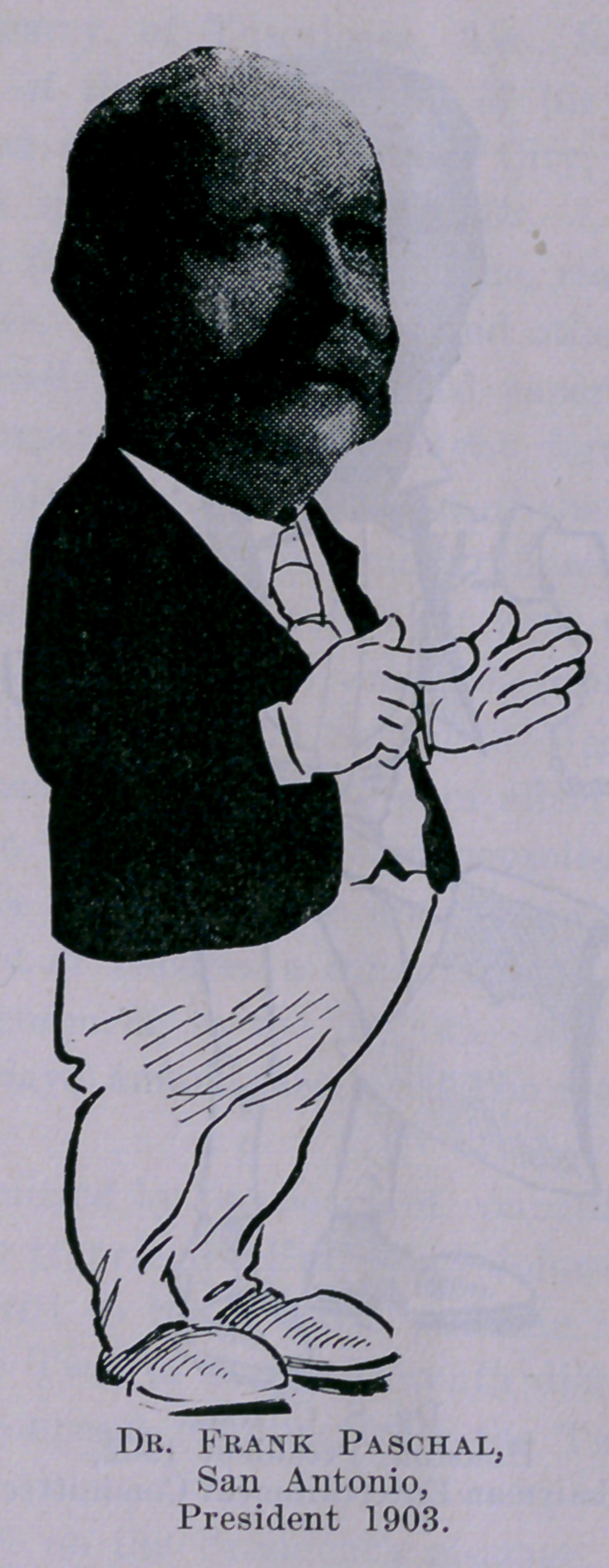


**Figure f16:**
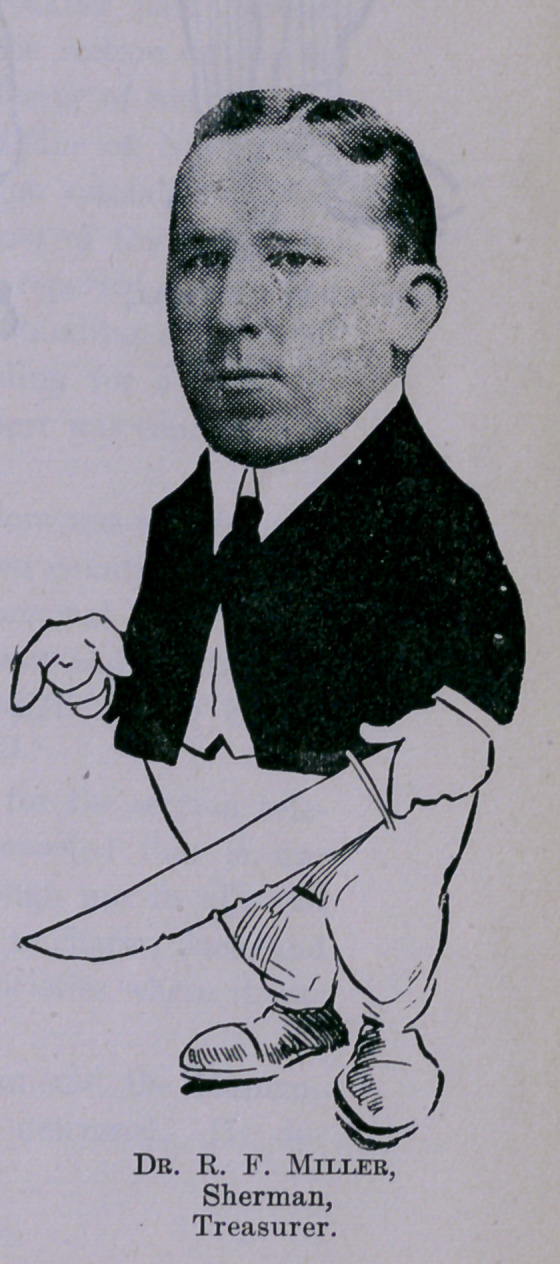


**Figure f17:**
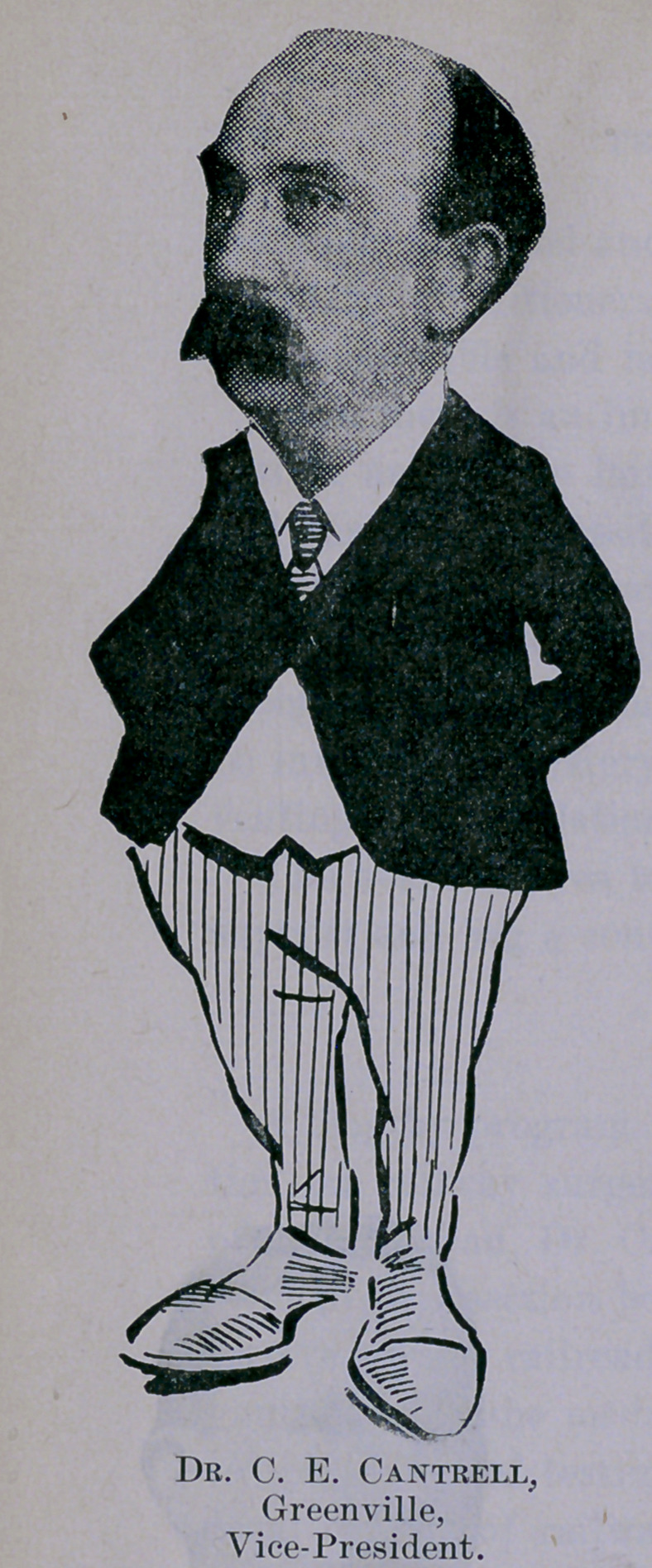


**Figure f18:**
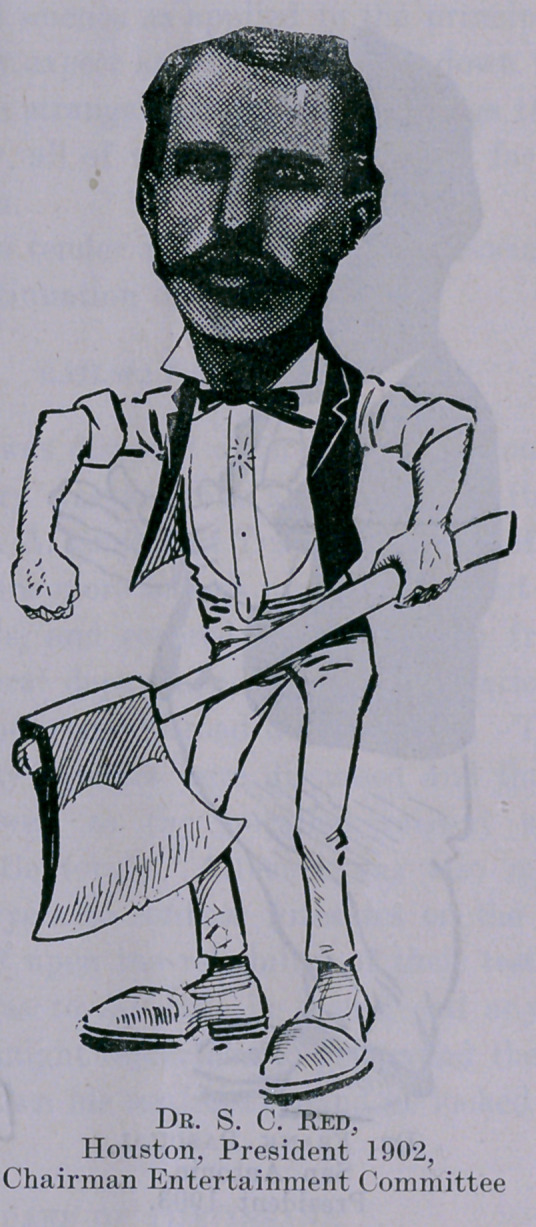


**Figure f19:**
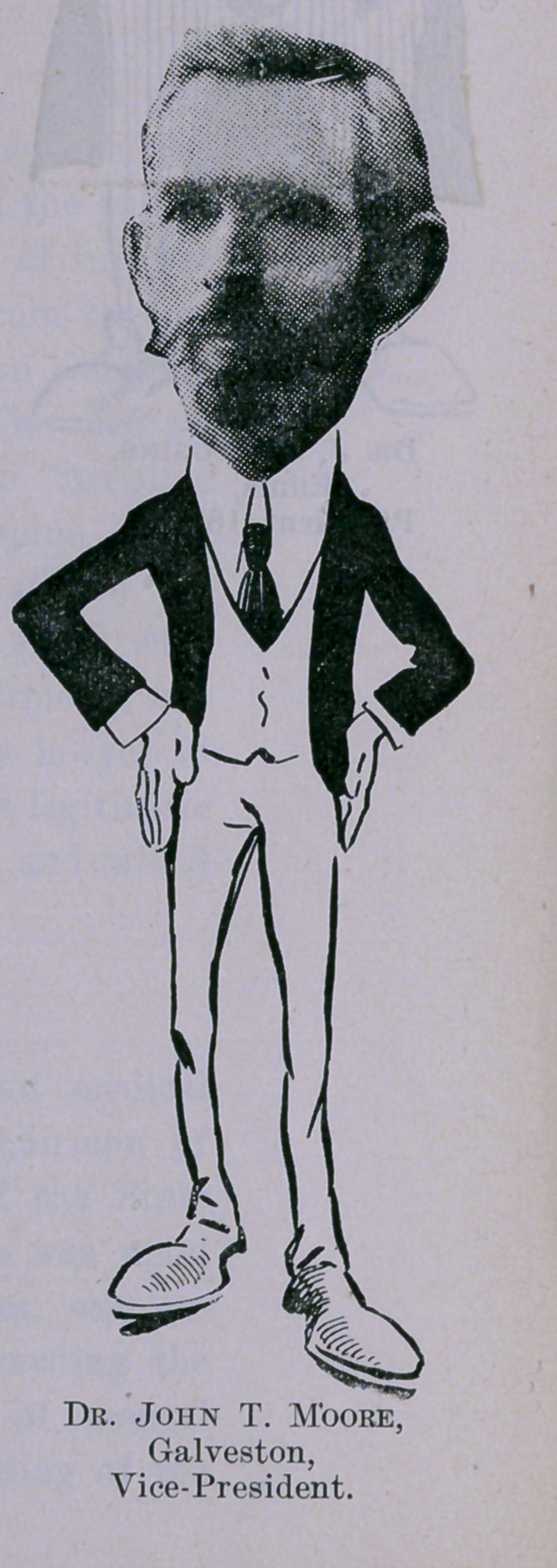


**Figure f20:**
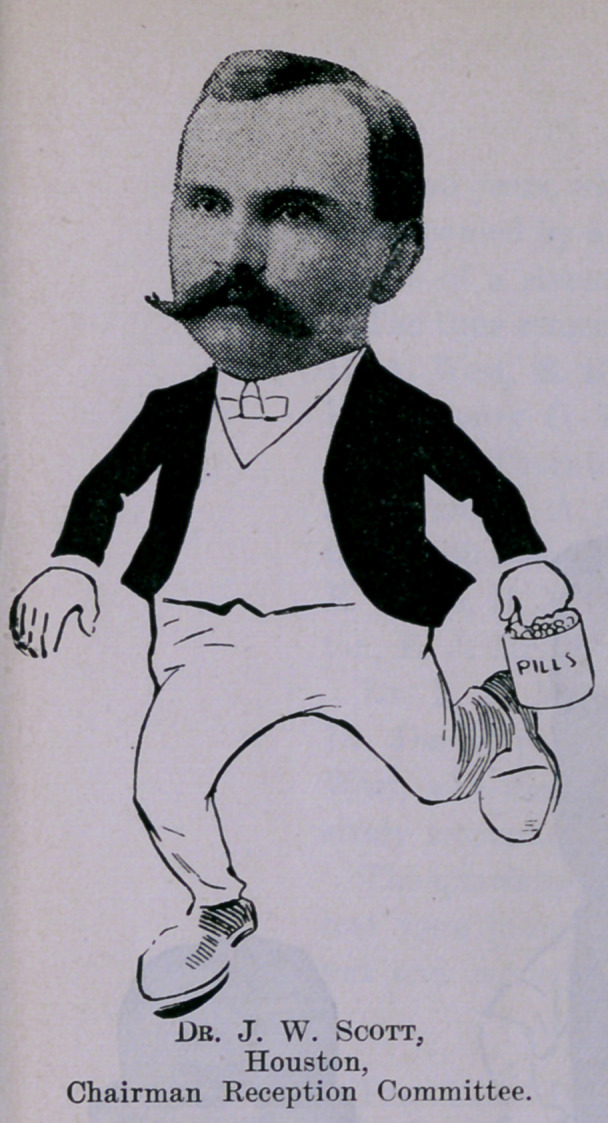


**Figure f21:**
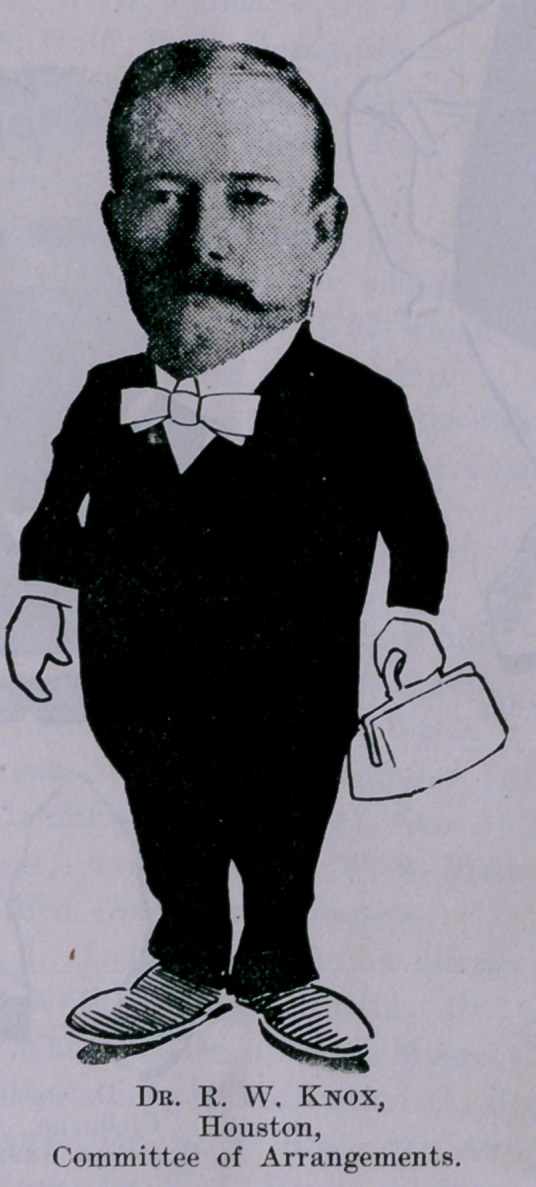


**Figure f22:**
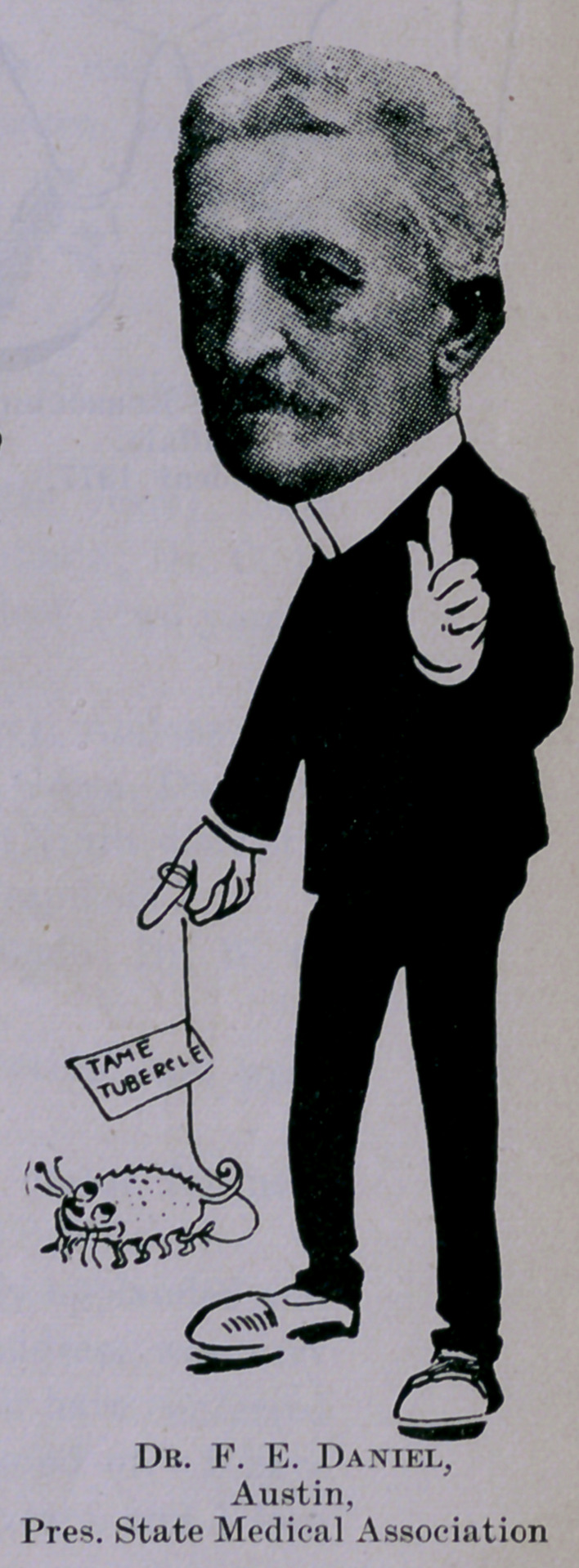


**Figure f23:**
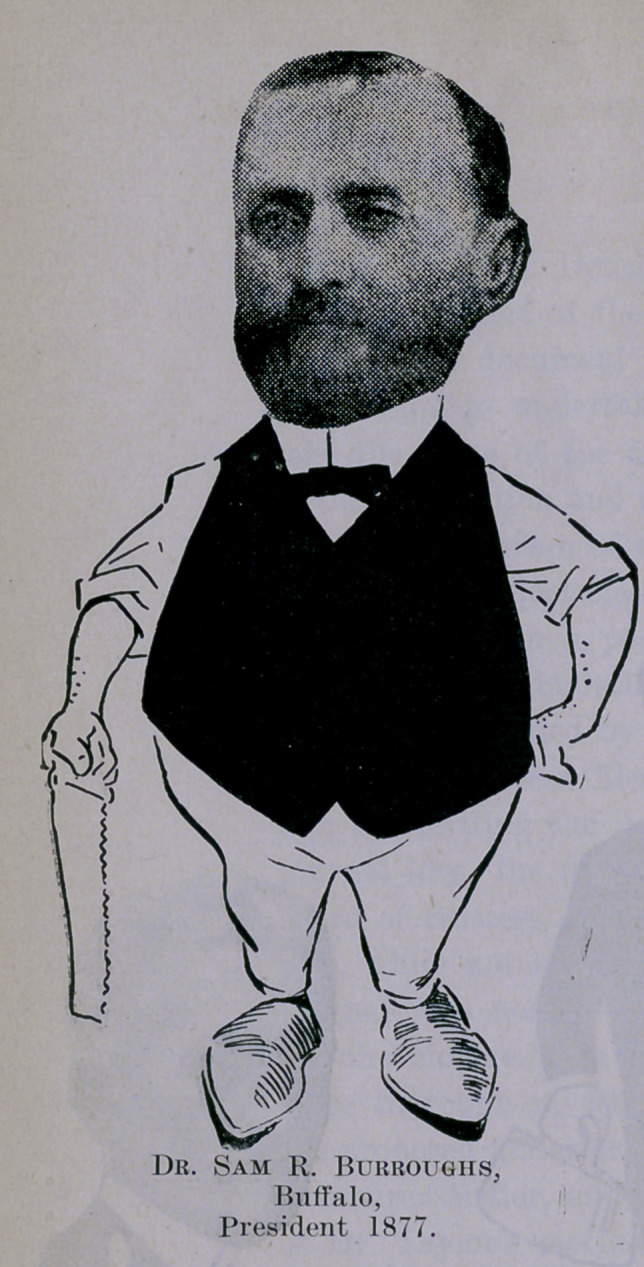


**Figure f24:**
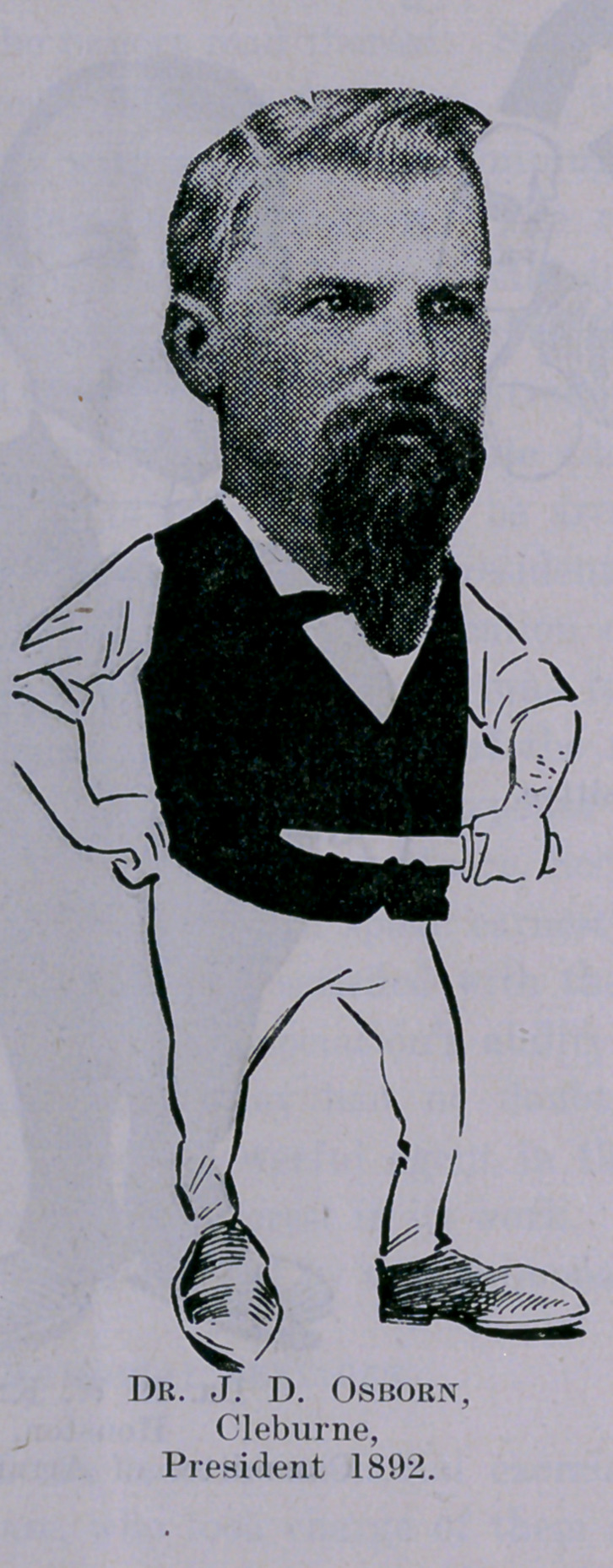


**Figure f25:**
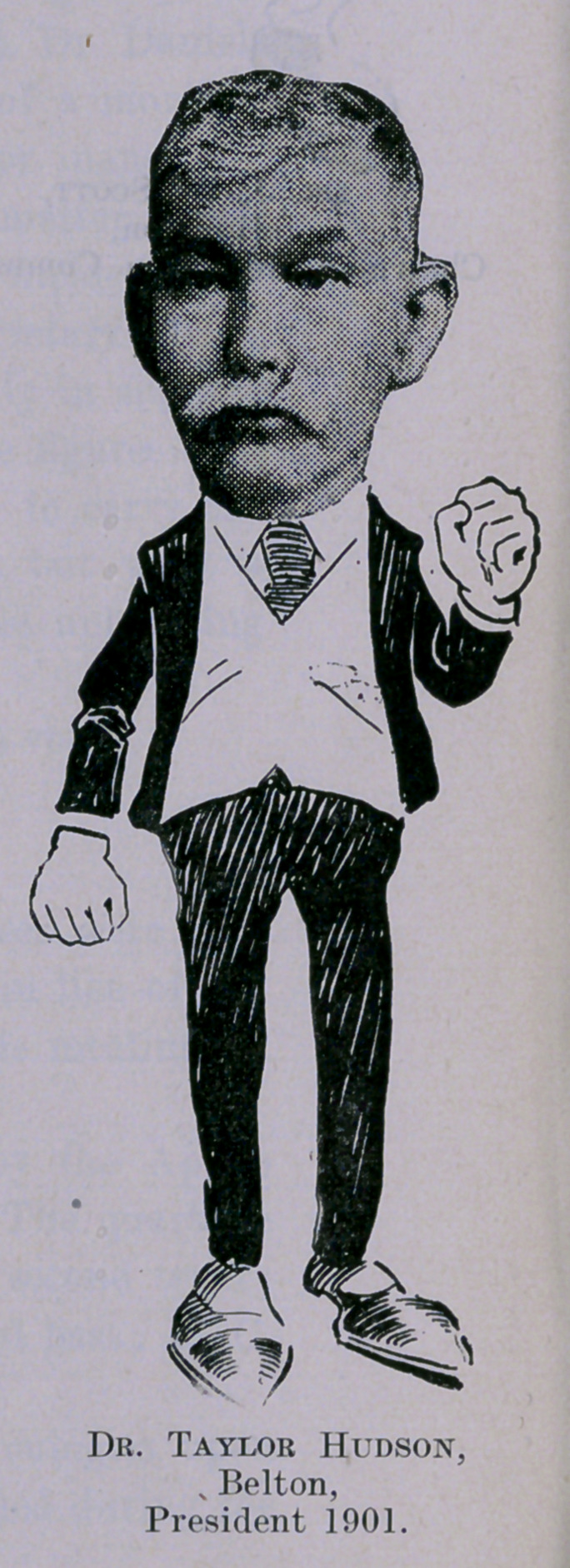


**Figure f26:**
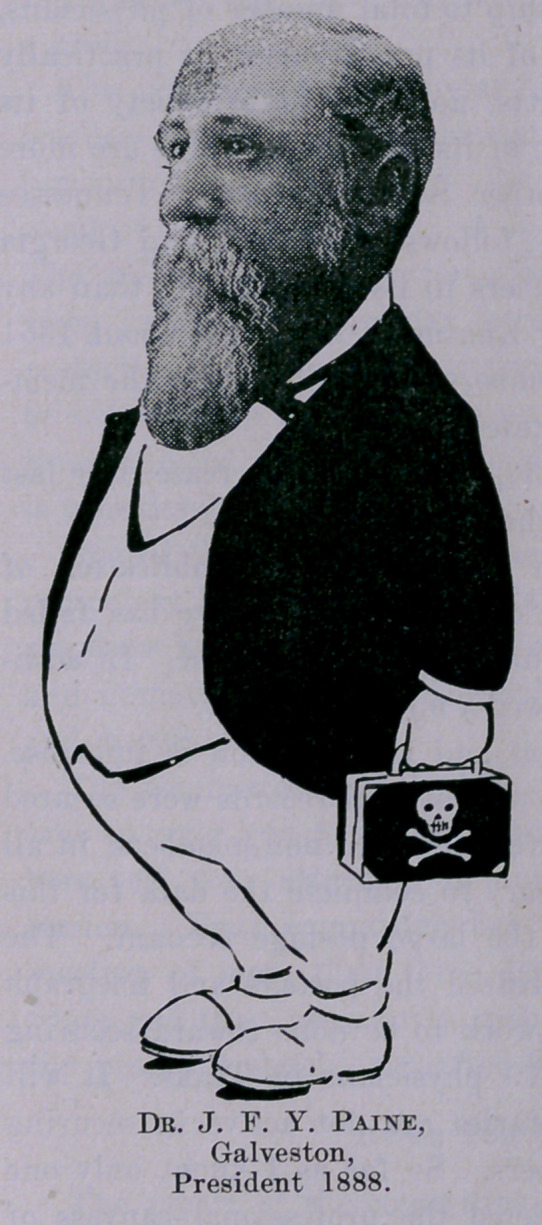


**Figure f27:**